# Depressive symptoms in elderly participants of an open university for elderly

**DOI:** 10.1590/S1980-57642011DN05020005

**Published:** 2011

**Authors:** Samila Sathler Tavares Batistoni, Tiago Nascimento Ordonez, Thaís Bento Lima da Silva, Priscila Pascarelli Pedrico do Nascimento, Priscilla Tiemi Kissaki, Meire Cachioni

**Affiliations:** 1 Professor, PhD in Gerontology, School of Arts, Sciences and Humanities of the University of São Paulo, São Paulo SP, Brazil; 2 Graduate in Gerontology, School of Arts, Sciences and Humanities of the University of São Paulo, São Paulo SP, Brazil; 3 Graduate in Gerontology and reading for Masters at the Department of Neurology of the University of São Paulo School of Medicine, São Paulo SP, Brazil; 4 Undergraduate Student in Gerontology, School of Arts, Sciences and Humanities of the University of São Paulo, São Paulo SP, Brazil

**Keywords:** aging, depression, university of the third age

## Abstract

Although the prevalence of depressive disorders among the elderly is lower than among the younger population, the presence of significant symptoms of depression is common in this group. Studies report that participation in social, educational and leisure activities is related to fewer depressive symptoms in this population.

**Objective:**

The aim of this study was to examine the prevalence of depression among elderly participants of an Open University for the Third Age, in terms of the time studying.

**Methods:**

The study had a cross-sectional design and the participation of 95.2% (n=184) of total enrollers in the first half of 2010 on the activities of the Third Age Open University’s School of Arts, Sciences and Humanities of the University of São Paulo. All participants answered a socio-demographic questionnaire and the Geriatric Depression Scale (GDS-15).

**Results:**

An association between studying time of over one semester at the University of the Third Age and a lower rate of depressive symptoms, was observed.

**Conclusion:**

Study time of over one semester was associated with less depressive symptoms, acting as a possible protective factor against depression.

## Abstract (pt)

Embora a prevalência de transtornos depressivos entre idosos seja menor do que entre a população mais jovem, a presença de sintomas significativos de depressão é frequente nesse grupo. Estudos documentam que a participação em atividades sociais, educacionais e de lazer se relacionam com a diminuição de sintomas depressivos nessa população.

**Objetivo:**

Este estudo examinou a prevalência de sintomas depressivos entre idosos participantes de uma Universidade Aberta para a Terceira Idade, tendo como referencial o tempo de participação.

**Métodos:**

O estudo teve um delineamento transversal, com a participação de 95,2% (n=184) do total dos inscritos no primeiro semestre de 2010 nas atividades da Universidade Aberta à Terceira Idade da Escola de Artes, Ciências e Humanidades da Universidade de São Paulo. Todos responderam a um questionário sócio-demográfico e à Escala de Depressão Geriátrica (GDS-15).

**Resultados:**

Observou-se a associação entre o tempo de participação superior a um semestre na Universidade da Terceira Idade e menor índice de sintomas depressivos.

**Conclusão:**

O tempo de participação superior a um semestre letivo associou-se com menor sintomatologia depressiva, atuando como um possível fator protetor contra a depressão.

## Introduction

Although the majority of older adults have a good level of well-being in later life, the heterogeneity of aging also allows the possibility of aging with significant negative emotional changes. Experiencing depression in old age is one such possibility. Although the prevalence of this type of clinically diagnosed mood disorder is lower among the older population than in younger groups (around 3% of elderly versus 5% among the general population), the presence of depressive symptoms remains significant and higher than occurs in some other age groups.^1^ Studies show that between 15 and 34% of community dwelling elderly exhibit a substantial number of depressive symptoms which, while not meeting clinical criteria for depression diagnosis, are significantly associated with deleterious effects on several domains of physical, psychological, cognitive and social functioning.^2^

The presence of depressive symptoms in late life is associated with greater incapacity for activities of daily living and worst quality of life indices with comorbidities, greater medication use, a higher number of life stressing events, higher mortality, poorer self-reported health and less perceived social support.^3^ Depression also impacts health-related behavior such as adherence to medical treatment, leading to a chronic worsening of physical diseases, raising the risk of comorbidity and mortality. Suicide rates are also high among older adults with depressive symptoms.^4^

The symptoms of depression, although more unstable in later life than during other times, may become a chronic or recurrent condition in around 50% of older adults affected, especially among ailing elderly.^5^ Due to the reduced chance of aging well, increased family burden and higher healthcare utilization associated with depression, the World Health Organization has proposed that the prevention and treatment of depression be made a public health priority.^6^ These actions are based on evidence that physical, psychological and psychosocial interventions can be effective for treating late-life depression and for promoting emotional health during the aging process.^7^

Risk factors for developing depressive conditions in older adults include sociodemographic variables such as advance age, female gender, low schooling, limited means and, independent of marital status, absence of a partner.^8-11^ Stressing life events, social isolation, or physical diseases have also been implicated as factors contributing to the development of depression in older adults.^10-14^ It is important to point out that one factor alone is insufficient to promote the emergence of depressive pictures, but an accumulation and interaction of these factors render elderly individuals more susceptible to the condition.^14^

Some studies have reported that participation in social, educational and leisure activities exert a protective effect^15,16^ and even serve as an effective treatment for depression.^17,18^ However, there is no consensus in the literature regarding the mechanisms involved in the relation between depressive symptoms and socioeducational and leisure activities. Some theories center on the opportunities that these activities provide, such as the possibility of reducing isolation through the development of a support network,^18-20^ positive influence on the beliefs and attitudes surrounding old age, and an increased sense of well-being and quality of life.^21-23^

In Brazil, numbering among the modalities of socioeducational and leisure activities on offer to older adults, are the programs run by the Open University for the Third Age. This is a modality of permanent education involves University level, multidisciplinary further education activities aimed at mature and older adults. These activities are grounded in the premise that taking part in intellectual, physical, social, cultural, artistic and leisure activities promote health, psychological and social well-being as well as citizenship of this client group, known generically as the Third Age.^22^

However, scant data or studies are available in the literature related to the emotional health and depressive symptomatology of the elderly who take part in activities based on this model of permanent education. A broad literature search of the SCIELO database spanning the maximum period available, retrieved only eight studies that sought to identify the prevalence of depressive symptoms among elderly participants of socioeducational and leisure activities.^19,20,23-28^ Of these studies, five were carried out in older adults who frequented the Open University for the Third Age.^19,20,23,25,27^

One of these studies, by Carneiro et al.,^19^ reported that deficits in social skills appear to constitute a factor of susceptibility to low quality of life and depression in elderly individuals. Also, in a cross-sectional, descriptive, epidemiological study of a sample of elderly participants of the Open University for the Third Age of the Federal University of Pernambuco (UnATI UFPE), Leite et al.^25^ identified cases of depression in almost a quarter of the sample, a level considered high compared to international studies conducted in communities of elderly.^29^

By contrast, a study in a sample of 103 participants of an Open University for the Third Age (UnATI) by Irigaray and Schneider^20,23,27^ showed that it was possible to age well in the absence of depressive symptoms. The authors suggested an association between UnATI participation time of over one year and a lesser degree of depression, as well as improved perceived quality of life.

In a study performed in Spain which sought to quantify the independence of elderly residents of an urban area and to identify the risk factors involved in their deterioration, noted that participation in groups was an external or environmental factor which acted as a mechanism delaying functional dependence.^30^ Thus, elderly who were more socially engaged delayed the emergence of functional dependence and depressive symptoms. In addition, a significant association between depressive symptoms and functional capacity was also found, i.e. the greater the conservation of functional capacity, the lower the presence of depressive symptoms.^30^ It can therefore be inferred that engagement in social activities promotes physical, mental as well as intellectual, functioning.

Against this background, the aim of the present study was to investigate, based on studying time, the prevalence of depressive symptoms in a sample of mature and older adults studying at the Open University for the Third Age at the School of Arts, Sciences and Humanities of the University of São Paulo (EACH USP). This program was recently implemented, during the second semester of 2006, in the Eastern district of the municipality of São Paulo.

## Methods

### Participants

The sample comprised 184 mature and older adults of both genders older than 50 years of age. Study subjects were enrolled on the first semester of the 2009 academic year at the School of Arts, Sciences and Humanities of the University of São Paulo. After assessment, the participants were stratified into two groups: Group I, 89 recently-enrolled students, having frequented the University for less than one academic semester; and Group II, 95 students who had frequented the University for longer than one academic semester.

### Study site

The University of São Paulo (USP) set up an Open University for the Third Age in 1993 following approval and acceptance of a proposal made to the Pro-Rector for Culture to offer a university course to elderly individuals. The prerequisites for enrollment on the courses include a minimum age of 60 years (except for workshops and talks, whose excess places are offered to individuals aged 50 or older), résumé assessment, interview or university degree. No certificates of course completion are offered in connection with the program nor does enrollment entitle participants to take part in regular courses offered by USP. The curriculum is split into three areas:

1. **Regular disciplines:** through places on graduate courses;
2. **Supplementary didactic-cultural activities:** mini-courses and workshops;
3. **Supplementary physical-sports activities:** rambles and physical fitness exercises.

The program is run across several campii and units of USP plus the cities of Bauru, Piracicaba, Pirassununga, Ribeirão Preto and São Carlos and in São Paulo at the University Campus and the School of Sciences and Humanities (EACH). The Open University for the Third Age of the EACH-USP is a permanent-education program for refreshing and acquiring knowledge. The program aims to promote the health, psychological and social well-being, as well as citizenship, of participants. Through workshops, lectures and disciplines of the graduate courses offered every semester, the elderly participants, predominantly women from the Eastern region of São Paulo who had studied only to primary school level, break paradigms and stereotypes related to late-life and the aging process. The elderly participants also exchange experiences through the intergenerational contact established among the different generations present in the University setting.^31^

Since its inception, the number of activities offered at the Open University for the Third Age of the EACH-USP has gradually increased from 8 in 2006 to 25 in 2009, while new courses have been added and teaching staff have joined the Program, as shown in Chart 1.

**Chart 1:** Activities offered in 2009.

Activity			Course from unit
Supplementary didactic-cultural activities			
	1.	Workshop on promoting health and quality of life	Gerontology
	2.	Cinema and family: the role of the elder	Gerontology
	3.	Series of debates: age limit on sex?	Gerontology
	4.	Workshop challenging memory	Gerontology
	5.	Social tourism - living in São Paulo	Leisure and tourism
	6.	Workshop: conversations about the ethics of life: learning about bioethics	Gerontology
	7.	Workshop providing guidance on correct use of medicines	Gerontology
	8.	Workshop on emotional health	Gerontology
	9.	Active aging	Gerontology
	10.	Workshop on healthy eating	Gerontology
	11.	Project tell your story	Leisure and tourism
	12.	Volleyball for the third age	Sciences of physical activity
	13.	Action for the prevention of falls in elderly	Sciences of physical activity
	14.	Sciences of the earth and sustainability	Sciences of nature degree
	15.	Elderly online	Gerontology
	16.	Meeting of generations and information technology	Gerontology
	17.	Talk: our dream of every day	Gerontology
	18.	Talk: risk management in aging	Gerontology
	19.	Talk: accessibility and universal design	Gerontology
	20.	Talk: the big issue of accessibility to medications	Leisure and tourism
	21.	Talk: third age: rights and duties	Leisure and tourism
Regular disciplines			
	1.	Fundamentals of law	Leisure and tourism
	2.	Psychological care practices in the aged	Gerontology
	3.	Pathological processes in aging I	Gerontology
	4.	Cultural resources and historical heritage in leisure and tourism	Leisure and tourism
	5.	Inventory of leisure and tourism	Leisure and tourism
	6.	Policy and programs for healthcare in the elderly	Gerontology

### Instruments

The sociodemographic data were collected using a questionnaire including the following variables: gender, age, schooling, marital status, family income, occupation (worked and/or retired) and time studying at the UnATI EACH-USP (in semesters). The Geriatric Depression Scale - GDS-15: the GDS is one of the most commonly used measures for screening depression in the elderly population. In the present study, a brief version of the instrument in Portuguese consisting of 15 questions with answers classified as yes or no, and a cut-off point of 5/6 (non-case/case), was adopted. Total score on the GDS is calculated based on the sum of the responses and indicates extent of depressed mood, with 0 being the lowest score and 15 the highest. The version used was adapted from Yesavage et al.,^32^ and is considered a valid and reliable scale for use in Brazilian samples.^33^

### Procedures

The present study is part of the research project, “Permanent Education - Benefits of the Open University for the Third Age - EACH USP”, funded by the Anísio Teixeira National Institute of Educational Studies and Research - Ministry of Education (Selection Process nº 02/2009 - INEP/MEC). The aim of this prospective study was to identify the characteristics and benefits of participation of elderly in an Open University for the Third Age, based on data collected by previously trained examiners.

### Ethical aspects

The research project was approved by the Research Ethics Committee of the Psychology Institute of the University of São Paulo, under report number 2010.043. The present study was conducted in accordance with Resolution nº 196/96 on the Directives and Regulatory Norms on Research in Humans (National Board of Health, 1996), and participants signed an Free and Informed Consent Term. Individuals who agreed to take part in the study signed the Free and Informed Consent Term and were included in the study population. All participants were provided with a guarantee of secrecy of information, confidentiality and privacy.

### Statistical analyses

In order to describe the profile of the sample based on the variables under study, frequency tables were constructed for the categorical variables, while descriptive statistics were produced, such as measures of position and dispersion, for the continuous variables. Kolmogorov-Smirnov test was used to identify the absence of a normal distribution for the continuous variables (p<0.05) and consequently these variables were treated by applying non-parametric tests. Mann-Whitney test was therefore used to compare continuous variables between the two groups.^34,35^

The Chi-square test was used to compare the categorical variables between the groups and, whenever 3 categories or more needed analyzing, the Chi-square test for multiple samples was employed^34-36^. The data were double keyed into version 3.1 of the Epidata Program and were validated using the validate mode. Statistical analyses were performed using the Statistica 7.0^36^ and SPSS v.17 computer programs. A 5% level of significance was adopted for the statistical tests.

## Results

The sociodemographic characteristics of the study sample are shown in Table 1. The sample comprised 184 participants between 50 and 80 years of age, with the majority (56.5%) in the 60-69 year age group. With regard to marital status, most of the elderly were married (49.46%), and had completed high-school education. In terms of income, the participants had a heterogeneous profile. Of the 184 interviewees, 80.89% were retired. For time studying at the University, 14.13% had studied at the UnATI EACH-USP for one semester.

**Table 1:** Sociodemographic profile of participants overall and by group (n=184).

Variable	Overall			Groups						p-value
				Group I			Group II			
	n	%		n	%		n	%		
Gender										
Male	48	26.09		27	30.34		21	22.11		
Female	136	73.91		62	69.66		74	77.89		0.270^a^
Age groups (in years)										
50-59	31	16.80		19	21.35		12	12.63		
60-69	104	56.50		47	52.81		57	60.00		
70-79	46	25.00		21	23.60		25	26.32		
80+	3	1.60		2	2.25		1	1.05		0.309^b^
Marital status										
Single	24	13.04		12	13.48		12	12.63		
Married	91	49.46		44	49.44		47	49.47		
Separated	9	4.89		3	3.37		6	6.32		
Divorced	10	5.43		7	7.87		3	3.16		
Widow (er)	45	24.46		22	24.72		23	24.21		
Common-law marriage	5	2.72		1	1.12		4	4.21		0.503^c^
Schooling										
Illiterate	2	1.09		1	1.12		1	1.05		
Primary school education (not concluded)	28	15.22		13	14.61		15	15.79		
Primary school education (concluded)	16	8.70		7	7.87		9	9.47		
High school education (not concluded)	14	7.61		5	5.62		9	9.47		
High school education (concluded)	68	36.96		29	32.58		39	41.05		
University level education (not concluded)	9	4.89		5	5.62		4	4.21		
University level education (concluded)	47	25.54		29	32.58		18	18.95		0.087^b^
Retired										
No	35	19.02		20	22.47		15	15.79		
Yes	149	80.98		69	77.53		80	84.21		0.413^a^
Family income										
Up to 1 minimum wage	8	4.35		3	3.37		5	5.26		
From 1 to 2 min. wages	38	20.65		19	21.35		19	20.00		
From 2 to 3 min. wages	33	17.93		13	14.61		20	21.05		
From 3 to 4 min. wages	35	19.02		13	14.61		22	23.16		
From 4 to 5 min. wages	25	13.59		15	16.85		10	10.53		
From 5 to 10 min. wages	33	17.93		20	22.47		13	13.68		
Over 10 min. wages	12	6.52		6	6.74		6	6.32		0.177^b^
Lives with?										
Alone	42	22.83		17	19.10		25	26.32		
With spouse only	38	20.65		15	16.85		23	24.21		
With children	21	11.41		12	13.48		9	9.47		
With spouse and children	46	25.00		22	24.72		24	25.26		
With spouse, children and grandchildren	12	6.52		9	10.11		3	3.16		
Children live with you	2	1.09		1	1.12		1	1.05		
Children and grandchildren live with you	10	5.43		7	7.87		3	3.16		
Another person lives with you	13	7.07		6	6.74		7	7.37		0.293^c^
Previously studied at UnATI EACH-USP?										
No	89	48.37		89	100.00		0	0.00		
Yes, for 1 semester	26	14.13		0	0.00		26	27.37		
Yes, for 1 Year	22	11.96		0	0.00		22	23.16		
Yes, for more than 1 Year	47	25.54		0	0.00		47	49.47		<0.001^b^

a Chi-square test;

b Mann-Whitney Test;

c Chi-square test for multiple samples.

In order to ascertain the benefits yielded by time studying at the UnATI EACH-USP, participants of this study were divided into two groups: Group I (n=89) containing recently enrolled students who had studied for less than one academic semester, and Group II (n=95) included students that had studied for more than one academic semester (Table 1).

The two groups were submitted to statistics tests to identify differences in socio-demographic data (Table 1). The analysis revealed the absence of any difference between the two groups (apart from time studying at the UnATI EACH-USP) and allowed results for studying time to be analysed in isolation.

In relation to depressive symptoms, of the 184 individuals interviewed, only 17 participants scored greater than 6 points on the Geriatric Depression Scale (GDS-15) - a rating indicating mild to moderate depressive symptoms. Of these high scorers, 12 belonged to Group I and 5 to Group II. Analysis of depressive symptoms in the groups stratified by time studying revealed that Group II, comprising students studying more than one academic year, presented with fewer depressive symptoms (p-value=0.017) as shown in Table 3.

**Table 3:** Comparison of group I and group II scores on the Geriatric Depression Scale.

Variable		Descriptive statistics						
		n	Mean	SD±	Min.	Median (q1-q3)	Max.	p-value
Depressive symptoms	Group I	89	3.16	2.49	0.00	2.00 (01-04)	11.0	0.017^a^
	Group II	95	2.32	1.70	0.00	2.00 (01-03)	10.00	
	Total	184	2.72	2.15	0.00	2.00 (01-04)	11.0	

a Mann-Whitney Test.

## Discussion

The participants in the study were stratified into two groups: Group I (less than one semester studying) and Group II (more than one semester studying). The respective groups were compared using statistical tests and proved statistically similar for sociodemographics. This comparison is important in the present study because evidence shows that sociodemographic variables such as age, gender, schooling, impoverished background, and having a partner, independent of marital status, exert an influence on depressive pictures.^9,10^ Therefore, the homogeneity of the groups in terms of these data allowed inferences to be drawn, since the significant variable between the two was only time studying at the UnATI.

Time studying at UnATI EACH-USP of greater than one semester proved a good predictor of positive and low indices of depressive symptoms, in-line with results found in studies by Irigaray and Schneider^20-23^ as well as Loures and Gomes.^37^ These studies reported a direct association between intensity of depression and participating in a course at an Open University for the Third Age.

Besides time studying, an important finding in the present study was the number of cases of individuals scoring above the cut-off point for depression, where only 5 (3.57%) subjects scored 6, a rating of mild symptomatology on the GDS-15. Overall, a low rate of depressive symptoms was found compared to levels described in previous studies. A study by Leite et al.^25^ in a sample of 358 elderly (312 women and 46 men), found depression cases in almost a quarter of the sample (24.02%) of participants of the Open University for the Third Age Program of the Federal University of Pernambuco (UnATI UFPE). Although the cited study employed a sound methodology which screened for depressive symptoms and correlated results with sociodemographic data, participants were not stratified according to time studying.

In addition to the variable depression variable examined, other studies have also reported the positive impact of taking part in socioeducational program among elderly on their physical and mental health, attitudes and social relationships.^31,38^ Cachioni^38^ noted that participation in a University for the Third Age instilled a sense of social worth and respect in students. Moreover, participants made educational progress which in turn resulted in greater self-confidence and self-efficacy, as well as improved cognitive performance and productivity. Along the same lines, Ordonez and Cachioni^31^ found that participation by elderly in an Open University can have a positive effect on the attitudes of elderly toward younger adults and vice-a-versa, thereby fostering healthy intergenerational contact. Also, this enabled discussion of the concepts and perceptions of a more positive late-life, free of prejudice, helping elderly to express their true needs and expose the importance of their participation in society.

This study corroborated the notion that participation in an activity in which the individual is encouraged to gain and maintain autonomy and independence is conducive to achieving a good quality of life. In this setting, participants have the opportunity to become better prepared to cope with the stressors of everyday life by expanding their social network, and emotional, information and instrumental support. Furthermore, the information and knowledge gained strengthen personal resources such as self-efficiency, social skills and problem solving.^23^

This study had some limitations. For instance the study did not include pre and post testing using a control group. Nevertheless, this study contributed to the Gerontology literature in that it involved mature elderly domiciled in the community who took part in socioeducational programs that may protect against depressive symptoms. In addition, the present study used scales previously adapted for the Brazilian elderly population. The current findings highlight that the maintenance of a good emotional state can lead to improved quality of life among the elderly because it allows them to remain active and independent for longer, and maintain a better preserved socially engaged life. Therefore, further studies are warranted on this increasingly important topic.

Future studies investigating depressive symptoms in participants of programs such as that run by the UnATIs should involve a larger number of subjects and include longitudinal follow-up in order to confirm long-term maintenance of improved emotional state. Such studies should also provide a more in-depth description of the sociodemographic aspects and mood status, and seek to determine those activities in UNATI settings that are most protective against the development of depression, and verify a possible relationship between this protection and the methodology of the activity or teacher involved. Finally, further studies on depression in elderly are underway investigating an association of the condition with variables such as cognitive performance, beliefs held about aging, and quality of life, among participants of Universities for the Third Age.

## Figures and Tables

**Table 2:** Distribution of scores on the Geriatric Depression Scale Overall and by group.

Variable	Overall			Groups				
				Group I			Group II	
	n	%		n	%		n	%
Score on GDS								
00	15	8.15		7	7.87		8	8.42
01	42	22.83		16	17.98		26	27.37
02	49	26.63		23	25.84		26	27.37
03	28	15.22		12	13.48		16	16.84
04	23	12.50		12	13.48		11	11.58
05	8	4.35		5	5.62		3	3.16
06	7	3.80		4	4.49		3	3.16
07	3	1.63		2	2.25		1	1.05
08	3	1.63		3	3.37		0	0.00
09	3	1.63		3	3.37		0	0.00
10	2	1.09		1	1.12		1	1.05
11	1	0.54		1	1.12		0	0.00

Cut-off score >6.
